# Anaphylaxis in Pregnancy in the United States: Risk Factors and Temporal Trends Using National Routinely Collected Data

**DOI:** 10.1016/j.jaip.2019.04.047

**Published:** 2019

**Authors:** Stephen J. McCall, Jennifer J. Kurinczuk, Marian Knight

**Affiliations:** National Perinatal Epidemiology Unit, Nuffield Department of Population Health, University of Oxford, Headington, Oxford, United Kingdom

**Keywords:** Anaphylaxis, Pregnancy, Cesarean section, Risk factors, aOR, Adjusted odds ratio, GBS, Group B streptococcus, *ICD-9-CM*, *International Classification of Diseases*, *Ninth Revision*, *Clinical Modification*, NIS, National Inpatient Sample

## Abstract

**Background:**

Anaphylaxis in pregnancy is an understudied, rare, and severe complication of pregnancy.

**Objective:**

To describe the incidence and temporal trends, and to identify potential risk factors for anaphylaxis-related hospitalizations while pregnant in the United States.

**Methods:**

All hospitalizations while pregnant and any anaphylactic reactions were identified using *International Classification of Diseases, Ninth Revision, Clinical Modification* codes from the National Inpatient Sample, United States, over the period 2004 to 2014. Annual incidence rates of anaphylaxis during pregnancy were calculated. Logistic regression models assessed risk factors for anaphylaxis during pregnancy, presented as odds ratios (ORs) and 95% CIs.

**Results:**

During the period 2004 to 2014, the incidence of anaphylaxis during pregnancy was 3.8 (95% CI, 3.4-4.2) per 100,000 hospitalizations while pregnant. The incidence did not statistically differ during the period 2004 to 2014. After adjustment, there were 3 factors that increased the odds of anaphylaxis during pregnancy: cesarean delivery (adjusted OR [aOR], 4.19; 95% CI, 3.28-5.35) compared with noncesarean delivery; history of an allergic reaction (aOR, 4.05; 95% CI, 2.64-6.23) compared with no history; and a black race (aOR, 1.57; 95% CI, 1.15-2.15) and other race (aOR, 1.69; 95% CI, 1.08-2.63) compared with white race.

**Conclusions:**

Despite increased rates of cesarean delivery in the United States and consequent drug administration, there was no evidence of an increasing trend in anaphylaxis. Cesarean delivery and history of an allergic reaction allow the identification of women at risk of anaphylaxis. Not all women had clear risk factors, and preparations should always be in place to ensure timely management if this uncommon event occurs.

***What is already known on this topic?*** The United Kingdom has an estimated incidence of anaphylaxis in pregnancy of 1.6 cases per 100,000 maternities, mainly caused by antibiotic and anesthetic drug administration. The use of antibiotics and anesthetic agents given to women during pregnancy is increasing.

***What does this article add to knowledge?*** The United States had double the incidence of anaphylaxis compared with the United Kingdom. There was no increasing trend in anaphylaxis in pregnancy over 10 years. Nearly all women affected had no history of drug allergies.

***How does this study impact current management guidelines?*** Clinical awareness of the risk of anaphylaxis should not be restricted to women with a history of drug allergies. All theaters and labor wards should have access to adrenaline for immediate administration.

## Introduction

Anaphylaxis is a potentially fatal systemic hypersensitivity reaction, which is rapid in onset.^1^ It is characterized by life-threatening airway, breathing, or circulatory problems often with skin or mucosal changes. It commonly occurs when allergens trigger an IgE-mediated cascade causing mast cell activation, resulting in an anaphylactic reaction.^2^ In addition, severe hypotensive reactions can occur through non–IgE-mediated mechanisms; however, these reactions are less common.^3^, ^4^, ^5^

There is very little information regarding the incidence of anaphylaxis in pregnancy globally. A single-state study in the United States suggested an incidence of 2.7 cases per 100,000 births, whereas a national study in the United Kingdom reported an incidence of 1.6 per 100,000 births.^6^, ^7^ It has been proposed that anaphylaxis is increasing in the general population although it still remains a very rare event.^2^, ^8^, ^9^ This may also be the case in pregnancy due to an increase in exposure to potential allergens; however, there are no published data available to assess this. The rationale for this hypothesis is that elevated rates of cesarean delivery globally will result in an increased exposure to anesthetic drugs and antibiotics during delivery, which are recognized allergens.^10^

Given the large sample size, the data collected in the National Inpatient Sample (NIS) in the United States are regarded as a good source for the study of rare diseases including temporal analyses.^11^ Previous research has focused solely on women with pregnancy-related anaphylaxis and has not included a comparison population to examine the potential risk factors for anaphylaxis.^6^, ^7^, ^9^, ^12^, ^13^, ^14^ The NIS offers an opportunity to compare the characteristics of those with and without anaphylaxis during hospitalizations while pregnant.

This study therefore aimed to describe the incidence and temporal trends in incidence, and to identify potential risk factors for pregnancy-related anaphylaxis using the NIS.

## Methods

### Setting and data source

This study used nationally representative hospital discharge data from the US NIS from 2004 to 2014.^15^, ^16^ The Nationwide Inpatient Sample was renamed to the National Inpatient Sample after the sampling frame was restructured in 2012 and for the purposes of this analysis these datasets will be described as the NIS. The National Inpatient Sample and the Nationwide Inpatient Sample forms part of the Healthcare Cost and Utilization Project, Agency for Health Care Research and Quality, and is the largest all-payer publicly available data set in the United States. From 2004 to 2011, the NIS was a 20% stratified sample of hospital discharges from US community hospitals and annually contained data on approximately 8 million hospitalizations from 1000 hospitals in 45 States. From 2012, the database was redesigned to contain a more representative sample of hospitals, which took into account urban-rural location, teaching hospitals, size, and patient-related factors. The NIS has been validated against the National Hospital Discharge Survey and the Medicare Provider Analysis and Review, which showed that basic epidemiology characteristics were similar between these large routine databases.^17^, ^18^

### Identification of women with related pregnancy hospitalizations

The study population was all women with a pregnancy-related hospitalization. First, hospitalizations of all women giving birth were identified using an enhanced method of identification by applying relevant *International Classification of Diseases, Ninth Revision, Clinical Modification* (*ICD-9-CM*) procedure and diagnosis codes^19^ (see Table E1 in this article's Online Repository at www.jaci-inpractice.org).

Second, hospitalizations at any point during pregnancy were identified using additional *ICD-9-CM* diagnosis codes including 630 (hydatidiform mole), 631(abnormal products of conception), 633 (ectopic pregnancy), and abortion (632, 634-639). Those individuals who were older than 55 years or younger than 12 years or who were coded as male were excluded from the sample.

### Identification of anaphylaxis

Women with anaphylaxis during pregnancy or at birth were identified using the following *ICD-9-CM* diagnosis codes: 995.0 (anaphylactic shock or reaction unspecified), 995.60 to 995.69 (anaphylactic reaction due to food), and 999.4 (anaphylactic reaction to serum). The codes to identify cases of anaphylaxis are consistent with those used previously.^20^ This analysis excluded hospitalizations without any *ICD-9-CM* diagnosis or procedure code.

### Covariates and characteristics

The NIS collects information about the following medical characteristics and sociodemographic factors: age, race, income (reported as the quartile classification of the estimated median income of residents in the patient's ZIP code), insurance status (Medicaid and Medicare, private insurance, self-pay, no charge, and other), and admission at the weekend. Hospitalizations with a cesarean section were identified using the *ICD-9-CM* procedure codes 74.0 to 74.99. Hospitalizations with a coded medical history of allergy were identified using the *ICD-9-CM* codes V14.0 (history of an allergy to a medical agent) to V15.09 (personal history of allergy, other than to medicinal agents) and V727 (diagnostic skin and sensitization test).

### Statistical analysis

The unit of analysis was individual hospitalization rather than per woman. To account for the sampling design, weights were applied to provide an estimate for the entire US population. Discharge weights were provided by Healthcare Cost and Utilization Project for each year. The incidence rate and univariable and multivariable analyses were weighted by the weights provided.

### Incidence

Both the incidence estimates from the NIS sample and the US population are presented. These were calculated per 100,000 pregnancy hospitalizations for the period 2004 to 2014 with 95% CIs. A test for trend in proportions was performed to assess whether there was a statistical difference in the incidence rate over time. In addition, yearly rates of anaphylaxis in pregnancy were estimated and the rate of anaphylaxis in pregnancy in those who had a cesarean delivery was also estimated. The risk of an anaphylactic reaction in women undergoing a cesarean delivery compared with a vaginal delivery is presented as an incidence rate ratio with 95% CIs.

### Risk factors for anaphylaxis

Unconditional logistic regression analysis was conducted to compare the characteristics, medical history, and management of those with and without anaphylaxis during a pregnancy-related hospitalization. The rationale for the inclusion for each risk factor is presented in Table E2 in this article's Online Repository at www.jaci-inpractice.org.

Collinearity was assessed by a Pearson correlation coefficient; no covariates were considered to be collinear (correlation coefficient <0.30). Only one interaction was tested, which was between cesarean delivery and race due to an *a priori* hypothesis that mode of delivery may be related to race, as a result of socioeconomic reasons. There was no statistically significant interaction between cesarean delivery and race (Wald test: *P* > .05). Age had a linear relationship with the outcome variable (anaphylaxis in pregnancy) so was included as a continuous variable.

The forward stepwise selection was used to identify variables for the final multivariable model. In the first instance, potential candidate variables were selected from the univariable analysis using a *P* value of less than .1 as a cutoff for inclusion. Each potential risk factor was modeled sequentially against anaphylaxis (yes/no). Variables were added sequentially into the model from the lowest level of significance first. A variable was included in the final model if it was associated with the outcome and improved the fit of the data, which was assessed by the adjusted Wald test (*P* < .05).

### Missing data

To account for missing data, a complete case analysis was used in the univariable and multivariable models for the NIS analysis. A proxy variable model was created to assess the impact of using complete case analysis in the NIS. A proxy category for each variable was created to include the missing data. A further examination of the missing data on race was undertaken on the basis of the results from the multivariable analysis. To assess the impact of the missing data on the final multivariable model, we used a sensitivity analysis using 2 separate models, which categorized the missing data of race into the “white category” and the “black category,” respectively.

## Results

### Incidence

**Figure E1:**
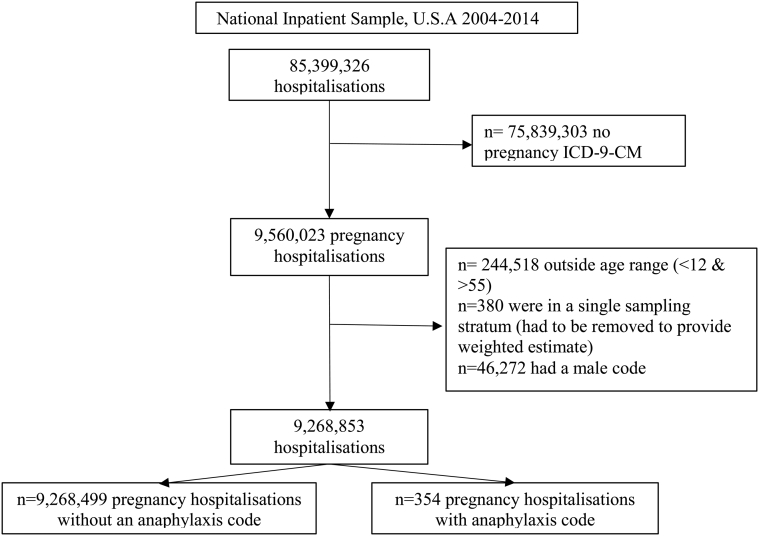
Derivation of study population from the NIS, United States.

The derivation of the study population is shown in Figure E1 in this article's Online Repository at www.jaci-inpractice.org. The overall incidence of anaphylaxis in pregnancy or at birth was estimated as 3.8 (95% CI, 3.4-4.2) per 100,000 hospitalizations while pregnant (Table I). The incidence of anaphylaxis in those who delivered by cesarean section was 7.7 per 100,000 cesarean deliveries (95% CI, 6.7-8.7). Two-thirds of anaphylactic reactions occurred in those with cesarean section. Those who had a cesarean section had nearly quadruple the risk of an anaphylactic reaction compared with those who did not (rate ratio, 3.8; 95% CI, 3.1-4.8). Because of the lack of granularity in the available variables, it is unclear whether the anaphylactic reaction occurred before or after delivery.

**Table I:** Incidence of anaphylaxis in pregnancy in the United States, 2004-2014

2004-2014	Unweighted	Weighted	Rate per 100,000 hospitalizations	95% CI	Unweighted		Weighted	
					Unadjusted incidence rate ratio	95% CI	Unadjusted incidence rate ratio	95% CI
Total number of hospitalizations	9,268,853	44,323,268						
Anaphylaxis in pregnancy	354	1,694	3.8	3.4-4.2				
No cesarean section	126	607	2.0	1.7-2.4	Reference		Reference	
In those with cesarean sections∗	228	1,086	7.7	6.7-8.7	3.9	3.1-4.8	3.8	3.1-4.8

∗ 2004-2014: 2,944,031 cesarean sections (unweighted).

**Figure 1:**
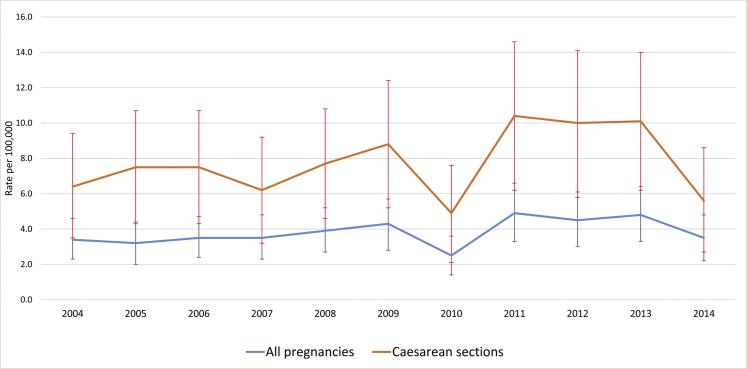
Trend in incidence of anaphylaxis in pregnancy per 100,000 pregnancy-related hospitalizations, 2004 to 2014.

The incidence of anaphylaxis in pregnancy remained between 3 and 5 per 100,000 hospitalizations while pregnant during the period 2004 to 2014 (Figure 1; Table E3, available in this article's Online Repository at www.jaci-inpractice.org) with the exception of 2010, when there was a nonsignificant reduction to 2.5 per 100,000 (95% CI, 1.4-3.6) hospitalizations while pregnant. For each year, the incidence of anaphylaxis in those who had a caesarean section was higher and followed a similar pattern to that of the rate of anaphylaxis in all pregnancies (Figure 1; Table E4, available in this article's Online Repository at www.jaci-inpractice.org).

### Risk factors for anaphylaxis in pregnancy

The characteristics of women and risk factors for anaphylaxis in pregnancy are presented in Table II. Women who had anaphylaxis during a pregnancy-related hospitalization tended to be older, have a “black” or “other” racial identity, have had a cesarean section during the hospitalization, received Medicaid, and had been admitted during a weekday. Only 2% of the NIS population had a code indicating a history of an allergy, while 8% of women with anaphylaxis had a history of an allergy (*P* < .05). There was no difference in the distribution of median household income according to anaphylaxis status.

**Table II:** Characteristics and risk factors for anaphylaxis in pregnancy-related hospitalizations in the United States, 2004-2014

Characteristic	No anaphylaxis in pregnancy (n= 9,268,499), n (% of nonmissing)	Anaphylaxis in pregnancy (n = 354), n (% of nonmissing)	Unadjusted odds ratio∗	95% CI	*P* value
Age (y), mean ± SD	28 ± 6.24	29 ± 6.07	1.02	1.01-1.04	.003
Race					
White	4,021,467 (52.2)	135 (45.9)	Reference		
Black	1,069,795 (13.9)	57 (19.4)	1.57	1.15-2.15	.005
Hispanic	1,787,349 (23.2)	58 (19.7)	0.94	0.69-1.29	.717
Asian or Pacific Islander	394,592 (5.1)	†		†	.102
Native American	60,938 (0.8)	†		†	.498
Other	371,882 (4.8)	21 (7.1)	1.62	1.04-2.52	.035
Missing‡	1,562,476	60			
Cesarean section					
Yes	2,943,803 (31.8)	228 (64.4)	3.84	3.09-4.78	<.001
No	6,324,696 (68.2)	126 (35.6)	Reference		
History of allergy					
Yes	185,721 (2.0)	28 (7.9)	4.13	2.76-6.18	<.001
No	9,082,778 (98.0)	326 (92.1)	Reference		
Primary expected payer					
Medicare	64,763 (0.7)	†	†		.31
Medicaid	3,936,464 (42.5)	174 (49.2)	Reference		
Private insurance	4,672,033 (50.5)	150 (42.4)	0.72	0.58-0.90	.003
Self-pay	310,077 (3.4)	16 (4.5)	1.14	0.70-1.86	.592
No charge	18,535 (0.2)	†	†		.808
Other	250,528 (2.7)	12 (3.4)	1.09	0.60-1.95	.784
Missing‡	16,099	†			
Admitted on a weekday					
Yes	7,469,510 (80.6)	307 (86.7)	1.61	1.19-2.19	.002
No	1,798,987 (19.4)	47 (13.3)	Reference		
Missing‡	†	0			
Median household income					
Lowest quartile	2,024,018 (27.5)	82 (28.3)	Reference		
Quartile 2	1,872,337 (25.4)	77 (26.6)	1.02	0.75-1.40	.895
Quartile 3	1,811,405 (24.6)	70 (24.1)	0.98	0.71-1.35	.889
Highest quartile	1,650,873 (22.4)	61 (21.0)	0.93	0.66-1.29	.649
Missing‡	1,909,866	64			

∗ Weighted to account for the complex sampling design.

† Small numbers suppressed because of NIS data disclosure requirements.

‡ Percentages were excluded.

The results of the multivariable unconditional logistic regression analysis are presented in Table III. After adjustment, 3 factors were statistically significantly associated with anaphylaxis in pregnancy. Women who had a history of an allergy had 4 times the odds of having an anaphylactic reaction (adjusted odds ratio [aOR], 4.05; 95% CI, 2.64-6.23) compared with women with no history of an allergy. Women who had a cesarean section had over 4 times the odds of having an anaphylactic reaction compared with women who did not have a cesarean section (aOR, 4.19; 95% CI, 3.28-5.35). The odds of anaphylaxis during a pregnancy-related hospitalization were 1.57 times higher in black women (aOR, 1.57; 95% CI, 1.15-2.15) and 1.9 times higher in women of “other” racial identity (aOR, 1.9; 95% CI: 1.08-2.63) compared with white women. The results for the proxy variable model and the 2 sensitivity analyses tended to attenuate results slightly but the results were not materially different.

**Table III:** Adjusted odds ratios for anaphylaxis in pregnancy-related hospitalizations in the United States, 2004-2014

Characteristic	Main model			Sensitivity 1∗			Sensitivity 2†			Sensitivity 3‡		
	Odds ratio	95% CI	*P* value	Odds ratio	95% CI	*P* value	Odds ratio	95% CI	*P* value	Odds ratio	95% CI	*P* value
History of allergy code												
Yes	4.05	2.64-6.23	<.001	3.90	2.60-5.87	<.001	3.83	2.55-5.75	<.001	3.93	2.61-5.92	<.001
Cesarean section												
Yes	4.19	3.28-5.35	<.001	3.78	3.04-4.70	<.001	3.77	3.03-4.69	<.001	3.79	3.05-4.72	<.001
Race												
White	Reference			Reference			Reference			Reference		
Black	1.57	1.15-2.15	.005	1.57	1.15-2.15	.005	1.48	1.10-1.99	0.010	1.38	(1.07-1.79)	.013
Hispanic	1.00	0.73-1.38	.977	1.00	0.73-1.37	.993	0.94	0.70-1.27	0.684	1.00	(0.73-1.37)	.991
Asian or Pacific Islander	1.58	0.97-2.57	.066	1.57	0.97-2.56	.068	1.48	0.92-2.38	0.109	1.57	(0.97-2.56)	.068
Native American	1.55	0.49-4.87	.452	1.55	0.49-4.85	.455	1.45	0.47-4.54	0.520	1.55	(0.49-4.85)	.455
Other	1.69	1.08-2.63	.021	1.68	1.08-2.63	.022	1.58	1.02-2.44	0.039	1.68	(1.08-2.63)	.022
Missing	—	—	—	1.24	0.91-1.70	.171	—	—	—	—	—	—

All models are adjusted for history of allergy, cesarean section, and race.

∗ Proxy variable model.

† Missing included in the white category of race.

‡ Missing included in the black category of race. All analyses were weighted to take into account the complex sampling design of NIS.

## Discussion

### Main findings

Over the period 2004 to 2014, there was no change in the incidence of anaphylaxis during a pregnancy-related hospitalization. Two-thirds of anaphylactic reactions during pregnancy were associated with cesarean sections. This study showed that cesarean section was associated with a nearly 4-fold increased risk of anaphylaxis during a pregnancy-related hospitalization. In addition, women who experienced anaphylaxis were more likely to have a history of allergic reactions and to be black or have an other racial identity.

### Findings in context

#### Incidence

These findings suggest that the incidence of anaphylaxis in pregnancy in the United States is more than twice that in the United Kingdom.^7^ It is possible however that the apparent higher incidence in the United States may be explained by misclassification within the routinely collected data; without verification from the medical records, it is impossible to identify how many, if any, cases were false positives. False-positive diagnoses were shown in a previous similar study of amniotic fluid embolism.^21^ Septic shock, thrombotic events, and coagulothopies caused by conditions such as amniotic fluid embolism and placental abruption may present similarly to anaphylaxis through cardiovascular compromise and cardiac arrest.

Alternatively, the higher incidence observed in the United States may represent a real phenomenon with the possible explanation being the routine bacteriological screening for group B streptococcus (GBS) carried out in the United States. This policy has increased the proportion of women receiving intrapartum antibiotics for GBS prophylaxis, which may have resulted in the higher incidence in the NIS sample compared with the United Kingdom,^22^ which uses a risk-based prevention strategy for GBS infection.^23^, ^24^

Another possible contribution to the observed higher incidence in the United States is a higher cesarean delivery rate compared with that in the United Kingdom. In the United States, 32% of births were by cesarean section during 2015^25^ while the rate was 28% in the United Kingdom during the period 2016 to 2017.^26^ As a result, more women in the United States would be exposed to anesthetic agents and prophylactic antibiotics before or after the cesarean section, thus placing a greater proportion of women in the United States at risk of an anaphylactic reaction compared with those in the United Kingdom. However, this 4% difference in cesarean section rate cannot completely explain the doubling of incidence between the United Kingdom and the United States, and so there must be other contributing factors for the difference in incidence.

Interestingly, there was no increase in the incidence of anaphylaxis in a pregnancy-related hospitalization during the period 2004 to 2014. This is contrary to our hypothesis, which postulated an increasing trend in anaphylaxis due to the increased incidence of cesarean section and consequent exposure to drugs, in particular antibiotics and anesthetic agents, during delivery.^10^ However, this may be because there was only a modest increase of 2% in cesarean deliveries during the period 2004 to 2014.

#### Cesarean section

Previous studies have shown that most women with anaphylaxis delivered by cesarean section; however, it was unclear whether this association with cesarean section was a cause or a consequence of the reaction.^6^, ^13^ A previous review recommended cesarean delivery in severe cases of anaphylaxis in order to deliver the infant immediately to reduce the risk of hypoxic brain injury.^13^ The findings using the US data show that women having a cesarean section had an increased risk of an anaphylactic reaction but there is no timing data so we cannot identify whether the cesarean and associated exposure was a cause or a management response to the reaction. A previous UK national study has shown that between 2012 and 2015 only 1 woman from the United Kingdom with anaphylaxis received antibiotics for GBS prophylaxis and then went on to deliver by cesarean section after the anaphylactic reaction.^7^ In contrast, approximately a third of reactions occurred immediately before a cesarean section and most of these were reactions to agents given as part of routine care before surgery.^7^ Because two-thirds of anaphylactic reactions in the United States were associated with cesarean section, it is likely that antibiotics and anesthetic agents made up a substantial component of the responsible causal agents. Other surgical intervention during the pregnancy would result in exposure to these agents; thus, cesarean section is unlikely to be unique as a cause of perioperative anaphylaxis.

#### Ethnicity

Our finding that anaphylaxis in pregnancy was higher for ethnic minorities is consistent with the wider body of literature on maternal mortality and morbidity. For instance, women from ethnic minorities have a higher maternal mortality rate than white women.^27^, ^28^, ^29^ There are a number of possible explanations for the relationship between anaphylaxis and black and other ethnic minorities. Black women are more likely to lack insurance coverage, present late to antenatal care,^29^ and are at an increased risk of a complicated pregnancy, which is reflected in an increased cesarean section rate compared to white women.^30^ Women who present late to antenatal care or are admitted in an emergency may provide an incomplete medical history, which may result in a drug or latex allergy being missed. The increased cesarean section rate and hence increased exposures to allergens in the black and other minorities may also be a partial explanation for this relationship. However, further work is required to describe the determinants for the racial disparity in anaphylaxis.

#### Previous known allergies

A previous study showed that women with anaphylaxis in pregnancy were more frequently recorded to have had previous allergic reactions than the background population.^7^ This study has furthered the evidence base by showing an association between a coded history of allergy and anaphylaxis in pregnancy. A history of an allergic reaction allows clinicians to identify women at increased risk of an anaphylactic reaction. We have no information in this study as to whether preventative measures were taken in relation to their specific allergies. However, more than 90% of women who experienced anaphylaxis did not have a previous drug allergy. Therefore, clinical awareness must not be restricted to those with a history of drug allergies. The National Audit Project on Perioperative Anaphylaxis in the United Kingdom found that the symptoms for anaphylaxis during pregnancy overlapped with those of other acute and severe obstetric morbidities, which could prevent the early recognition and timely management of anaphylaxis.^14^ The report stated that hypotension was the most objective physiological sign of anaphylaxis. As a result, anesthetists and obstetricians should be aware of the nonobstetric causes of hypotension in pregnant or postpartum women.^14^ However, this relationship may have been overestimated because the coding of previous drug allergies may have been more likely in those with an allergic reaction.

### Strengths and limitations

Use of a routine data source from the United States enabled the estimate of the incidence of this rare complication of pregnancy over a 10-year period in the whole of the United States. In addition, the NIS provided an ideal comparison population to allow the risk factors for anaphylaxis in this population to be explored although the extent of the risk factor analysis was limited to the variables available.

The inclusion of false positives into the NIS sample may have influenced the results of the risk factors analysis. Differential misclassification would have biased the results of this analysis if they were nonrandomly distributed between the categories of the risk factors. Random misclassification would have biased the point estimates toward the null.

The findings are only as reliable as the validity and sensitivity of the *ICD-9-CM* coding of the data. In particular, codes that are used more often or related to user payment would be well reported. The coding of anaphylaxis using *ICD-9-CM* may underreport the true number of cases of anaphylaxis.^31^ In particular, studies have shown that *ICD-9-CM* codes were only 66% sensitive in identifying this condition.^32^ Furthermore, there may have been other inaccuracies in the coding because 0.5% of the sample had a pregnancy code but were coded as male. In addition, the lack of a diagnosis code indicating a medical history does not exclude such a history due to the possibility of false negatives.

## Conclusions

Anaphylaxis in pregnancy is a very rare but serious event during pregnancy. There is no evidence of an increased trend in the United States despite increases in drugs given during iatrogenic delivery; however, the United States appears to have double the incidence of anaphylaxis compared with the United Kingdom. For the worst maternal and neonatal outcomes associated with anaphylaxis to be prevented, early diagnosis through recognition of hypotension and bronchospasm is required. In addition, not all women had clear risk factors and appropriate drugs and resuscitation equipment should always be in place to ensure timely management if this uncommon event occurs.

## Figures and Tables

**Table E1:** *ICD-9-CM* codes for delivery-related codes

V270-V279 (outcome of pregnancy) and 650 (normal pregnancy). In addition, maternities were identified using these procedure codes: 720, 721, 7221, 7229, 7231, 7239, 724, 726 (forceps); 7251, 7252, 7253, 7254 (breech extraction); 7271, 7279 (vacuum extraction); 728, 729 (other specified and unspecified delivery); 7322 (internal and combined version and extraction); 7359 (other manually assisted deliveries); 736 (episiotomy); and 740-749.9 (cesarean section). Diagnosis related group delivery codes were also used to identify maternities, and these codes were 370 (cesarean section) to 375 (vaginal delivery within operation room).

**Table E2:** Rationale for risk factors included in the US analysis

Potential risk factor	Rationale
Age	Demonstrated to be a risk factor for anaphylaxis.^E1^
Socioeconomic measures	Socioeconomic disparities have been documented in many areas in health including obstetrics. There is no reason to suspect this may be different with anaphylaxis.
Race	Racial disparities have been documented in many areas in health including obstetrics. There is no reason to suspect this may be different with anaphylaxis.^E2^
Cesarean delivery	Shown to be associated with anaphylaxis.E3, E4
Admission at weekend	Poor hospital outcomes have been associated with admission during weekends. There may be an increased risk of medical mistakes at weekend such as giving the wrong antibiotic to someone with an allergy.^E5^
History of allergy	Shown to be highly prevalent in those who have had anaphylaxis in pregnancy.^E4^

**Table E3:** Incidence of anaphylaxis in pregnancy-related admissions in the United States, 2004-2014

Year	Unweighted number of hospital records	Weighted number of hospital records	No. of anaphylaxis episodes	Weighted sample	Rate per 100,000 hospitalizations∗	95% CI
2004	894,589	4,181,660	31	143	3.4	2.3-4.6
2005	875,702	4,139,434	29	132	3.2	2.0-4.4
2006	895,518	4,214,792	32	149	3.5	2.4-4.7
2007	948,092	4,468,572	32	158	3.5	2.3-4.8
2008	888,796	4,146,832	35	163	3.9	2.7-5.2
2009	840,729	4,037,117	37	173	4.3	2.9-5.7
2010	796,916	3,809,515	19	94	2.5	1.4-3.6
2011	816,475	3,765,168	40	186	4.9	3.3-6.6
2012	770,955	3,854,776	35	175	4.5	3.0-6.1
2013	764,959	3,824,793	37	185	4.8	3.3-6.4
2014	776,122	3,880,610	27	135	3.5	2.2-4.8

∗ Weighted to provide national estimate.

**Table E4:** Incidence of anaphylaxis in pregnancy-related admissions in those with cesarean section in the United States, 2004-2014

Year	No. of cesareans	No. of anaphylaxis episodes in those with a cesarean	Weighted number of anaphylaxis episodes in those with a cesarean	Rate	95% CI	Rate ratio∗	95% CI	Weighted rate ratio∗	95% CI
2004	265,181	17	79	6.4	3.5-9.4	2.9	1.4-5.8	3.0	1.4-6.2
2005	266,595	21	95	7.5	4.3-10.7	6.0	2.7-13.5	5.8	2.6-12.8
2006	278,049	21	98	7.5	4.3-10.7	4.2	2.0-8.8	4.3	2.1-9.0
2007	305,139	18	89	6.2	3.2-9.2	2.7	1.3-5.4	2.7	1.3-5.6
2008	283,929	22	102	7.7	4.6-10.8	3.6	1.8-7.2	3.5	1.8-7.0
2009	275,427	25	117	8.8	5.2-12.4	4.3	2.1-8.5	4.2	2.0-8.9
2010	256,217	12	60	4.9	2.1-7.6	3.6	1.4-9.2	3.6	1.4-9.3
2011	265,445	28	127	10.4	6.2-14.6	4.8	2.5-9.5	4.5	2.3-8.8
2012	251,357	25	125	10.0	5.8-14.1	5.2	2.5-10.8	5.2	2.4-10.9
2013	248,025	25	125	10.1	6.2-14.0	4.3	2.2-8.6	4.3	2.2-8.6
2014	248,667	14	70	5.6	2.7-8.6	2.3	1.1-4.9	2.3	1.1-4.8

∗ The rate ratio comparing reactions in cesarean delivery to vaginal delivery.
